# A case of native valve endocarditis caused by *Burkholderia cepacia *without predisposing factors

**DOI:** 10.1186/1471-2334-11-114

**Published:** 2011-05-08

**Authors:** Hyun Kyun Ki, Sung Hea Kim, Seong Woo Han, Hae Suk Cheong

**Affiliations:** 1 Department of Medicine, School of Medicine, Konkuk University, Seoul 143-729, Korea

**Keywords:** Burkholderia cepacia, Endocarditis, Native valve

## Abstract

**Background:**

Infective endocarditis is rarely caused by *Burkholderia cepacia*. This infection is known to occur particularly in immunocompromised hosts, intravenous heroin users, and in patients with prosthetic valve replacement. Most patients with *Burkholderia cepacia *endocarditis usually need surgical treatment in addition to antimicrobial treatment.

**Case Presentation:**

Here, we report the case of a patient who developed *Burkholderia cepacia*-induced native valve endocarditis with consequent cerebral involvement without any predisposing factors; she was successfully treated by antimicrobial agents only.

**Conclusion:**

In this report, we also present literature review of relevant cases.

## Background

*Burkholderia cepacia *is a gram-negative bacillus. It is important nosocomial pathogen that particularly infects patients with cystic fibrosis [1] and chronic granulomatous diseases[2] and is known to be resistant to many anti-bacterial agents.

*Burkholderia cepacia *rarely causes endocarditis in community settings, but it is known to cause infective endocarditis particularly in intravenous heroin users, and in patients with prosthetic valve replacement [3]. However, infective endocarditis caused by *Burkholderia cepacia *in patients without predisposing factors is rare [4-6].

The treatment of *Burkholderia cepacia *endocarditis could be conservative, i.e., administration of antibacterial agents, and/or surgical modality [4-13]. According to the previous reports, most patients were prescribed trimethoprim-sulfamethoxazole and underwent valve surgery. Notwithstanding, the mortality in cases of infective endocarditis patients is not low despite the aggressive treatment of infective endocarditis [3].

Here, we report the case of a patient with *Burkholderia cepacia *endocarditis and cerebral involvement who had no predisposing factors, and the infection was successfully managed only by antimicrobial treatment. Along with this case, we also present a literature review.

## Case Presentation

A 77-year-old woman was transferred to our hospital from a local hospital for loss of consciousness and disorientation. She had experienced a fall 2 weeks before admission to our hospital. Plain spine radiography had revealed a spine compression fracture. Two days earlier, she was admitted to a local hospital for relief from back pain. However, during her stay at the hospital, she developed low grade fever, aphasia, and disorientation.

She was then transferred to our hospital for further evaluation of her symptoms that were suggestive of cerebral infarction. She denied any history of smoking, alcohol consumption, or intravenous drug use. She neither had cardiovascular disease nor exhibited any of the risk factors for it. Mild aphasia and hemianopsia were noted at neurological examination. Physical examination revealed a diastolic murmur at the mitral valve area.

**Figure 1:**
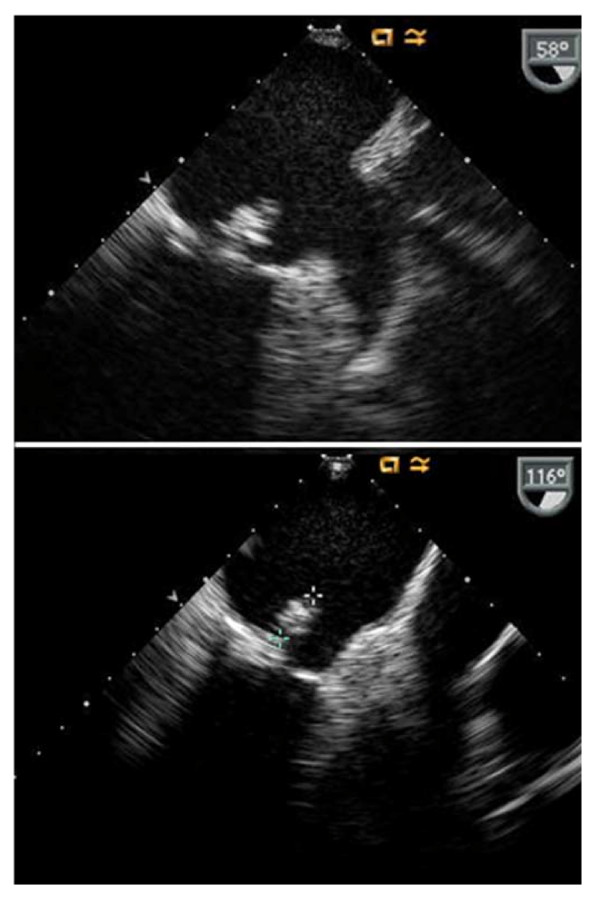
**A 13-mm echogenic mobile mass can be seen posterior to the mitral valve leaflet at 58° and 116° on the transoesophageal echocardiogram**. In addition, mitral stenosis (valve area = 1.7 cm^2^) with a thickened and partially mobile posterior mitral valve can be seen on the transthoracic echocardiogram.

**Figure 2:**
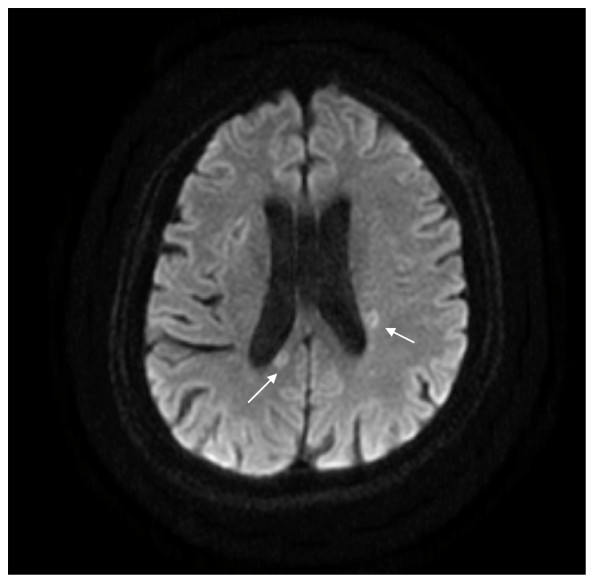
**Subacute and chronic infarction in both cerebral hemisphere and cerebellum**. High signal intensity on diffusion-weighted image (arrow).

The clinical manifestations were suggestive of mitral stenosis and cerebral infarction associated with emboli, which was considered the preliminary diagnosis. Echocardiography revealed mitral stenosis with a mobile 10-mm sized mobile mass (Figure 1). Brain magnetic resonance imaging (MRI) revealed subacute and chronic infarction in both the cerebral hemispheres and the cerebellum (Figure 2). On day 2 of hospitalization, fever above 38°C was noted; therefore, blood culture test was performed. She was administered ceftriaxone (2 g/d) and gentamicin (3 mg·kg^-1.^day^-1^) as an empirical treatment for infective endocarditis.

Despite the empirical antimicrobial treatment, intermittent low-grade fever was noted. Her blood culture was positive for *Burkholderia cepacia*, and antimicrobial susceptibility test yielded positive results for cefepime, ceftazidime, piperacillin, ciprofloxacin and trimethoprim-sulfamethoxazole (day 7 of hospitalization). Therefore, we changed the antimicrobial treatment to ceftazidime (6 g/d) from day 7.

Fever resolved completely from day 9, and blood culture was negative for *Burkholderia cepacia*. After 6 weeks of ceftazidime treatment, oral ciprofloxacin was prescribed for 2 weeks as an outpatient treatment.

After the antimicrobial treatment course was completed, infective endocarditis resolved completely; no relapse was noted during the follow-up period for 2 years.

## Discussion

*Burkholderia cepacia*, an opportunistic pathogen, is resistant to disinfectants and broad-spectrum antimicrobial agents. This pathogen often causes nosocomial infections in imunocompromised hosts, especially in patients with cystic fibrosis and chronic granulomatous diseases [3,4]. In addition, patients with cancer or chronic renal disease may also be susceptible to Burkholderia infection [14-16].

In these patients, central venous catheters are the primary source of bacteraemia [14]. Burkholderia bacteremia also noted in patients undergoing haemodialysis [15]. *Burkholderia cepacia *also causes infective endocarditis and skin and soft tissue pathology. This pathogen rarely causes infective endocarditis; however, there are some reports on the occurrence of this pathology in heroine addicts [4,10-13] and patients with prosthetic valves and valve replacements [3-5].

In our patients, *Burkholderia cepacia *had infected mitral valve despite the absence of predisposing factors that would make her susceptible to the infection. This case is different from the previous reports [3-13] in the following issues. First, most of the reported cases of *Burholderia cepacia *endocarditis involved the prosthetic valve. In contrast, native valve endocarditis was relatively less frequent [3,4,7-9]. *Burholderia cepacia-*induced native valve endocarditis has rarely been reported. As a predisposing condition, most patients with native valve endocarditis had a history of intravenous drug abuse [3,4,7-9] and mitral valve endocarditis has mostly been reported in patients having prosthetic valves [3-5]. However, our patient did not exhibit any of the predisposing factors related to this infection described previously.

Second, trimethoprim-sulfamethoxazole is commoly used for the treatment of this infection [4-9]. However, ceftazidime was administered to our patient because of cerebral involvement and risk of trimethoprim-sulfamethoxazole allergy. Brain MRI had revealed cerebral and cerebellar infarctions in our patient. These findings suggest that the emboli had migrated into the cerebral hemispheres and induced an ischemic change of the involved regions. Therefore, an antimicrobial agent, such as ceftazidime, that could penetrate the blood-brain barrier was required in this case.

Antimicrobial resistance of *Burkholderia cepacia *has posed a great challenge of treating the infection. This pathogen is intrinsically resistant to aminoglycosides and polymyxins. The antimicrobial effective against this pathogen are carbapenem, broad-spectrum beta-lactams (such as, piperacillin-tazobactam and ceftazidime), and trimethoprim-sulfamethoxazole. Therefore, because of such antimicrobial resistance, a combination of drugs and surgical treatment for valvulopathy is required [3-7,9]. However, we successfully treat the patient without the surgical treatment.

Third, replacement of the prosthetic or native valve has been required in most patients with infective endocarditis [3,7-9] but we did not need to perform any such surgery in our patient because of the excellent response to antimicrobial therapy. In addition, her valve function was also apparently preserved without surgery. On the bases of her valve function, life expectancy, and the morbidity involved in such surgical treatment, we decided against surgery and we were able to successfully treat the infective endocarditis by antibiotics only.

Fourth, in patients with *Burkholderia cepacia *endocarditis, the mitral valve is known to be less frequently involved than the tricuspid valve; mitral valve involvement has been reported in only those patients who previously had prosthetic valve replacement or valve repair surgery [3-5], and only 1 study reported aortic valve endocarditis without any predisposing factors [6].

In summary, we experienced a case of native valve endocarditis with cerebral involvement by *Burkholderia cepacia *without predisposing factors of Burkholderia infection, successfully managed by antimicrobial treatment only.

## Conclusion

Infective endocarditis is rarely caused by *Burkholderia cepacia*. Recently, we encountered a patient who developed *Burkholderia cepacia *endocarditis and cerebral involvement without the predisposing factors. We successfully treated the patient with only antimicrobial medication; surgical modality was not required.

## Consent

Written informed consent was obtained from the patient for publication of this case report and the accompanying images. A copy of the written consent is available for review by the Editor of this journal.

## Competing interests

The authors declare that they have no competing interests.

## Authors' contributions

HKK drafted this manuscript. SHK and SWH managed the patient. HSC revised this manuscript. All authors read and approved the final manuscript.

## Pre-publication history

The pre-publication history for this paper can be accessed here:

http://www.biomedcentral.com/1471-2334/11/114/prepub
